# The spread of antimicrobial resistance in the aquatic environment from faecal pollution: a scoping review of a multifaceted issue

**DOI:** 10.1007/s10661-025-13860-7

**Published:** 2025-03-25

**Authors:** Calum Cheung, Patrick J. Naughton, James S. G. Dooley, Nicolae Corcionivoschi, Cathy Brooks

**Affiliations:** 1 Nutrition Innovation Centre for Food & Health (NICHE), School of Biomedical Sciences, Ulster University, Coleraine, BT52 1SA UK; 2 Food Hygiene Unit, Bacteriology, Veterinary Sciences Division, Agri-Food Biosciences Institute (AFBI), Veterinary Sciences Division, BT4 3SD Belfast, Main UK

**Keywords:** Antimicrobial resistance, Antimicrobial resistance genes, Mobile gene elements, Wastewater treatment plant

## Abstract

Antimicrobial resistance (AMR) is a major global health concern accelerated by the misuse and mismanagement of antibiotics in clinical and veterinary settings, leading to longer treatment times, increased costs and greater mortality rates. The environment can play a major role as a source and disseminator of AMR, with faecal pollution, from both anthropogenic and non-anthropogenic sources making a significant contribution. The review aimed to identify how faecal pollution contributes to AMR in surface water, focusing on current methods of source tracking faecal pollution. The databases used were Medline Ovid® and Scopus. From the search, 744 papers from January 2020 to November 2023 were identified, and after the screening, 33 papers were selected that reported on AMR, aquatic environments and faecal pollution and were published in English. The studies were from six different continents, most were from Europe and Asia indicating faecal pollution is influenced by spatiotemporal differences such as population and sanitation infrastructure. Multiple different methodologies were used with a lack of standardised methods making comparability challenging. All studies identified AMR strains of faecal indicator bacteria showing resistance to a wide variety of antibiotics, particularly beta-lactams and tetracyclines. Few studies investigated mobile gene elements with class 1 integrons being the most frequently studied. Wastewater treatment plants were significant contributors, releasing large amounts of AMR bacteria into the environment. Environmental factors such as seasonal differences, temperature, rainfall and UV exposure, along with local antibiotic usage influenced the local resistome. Animals, both wild and domestic, introduced antimicrobial resistance genes and potential pathogens into the aquatic environment. Overall, faecal pollution is a complicated issue with multiple factors contributing to and facilitating the spread of AMR. Standardisation of methods and surveillance, robust wastewater management and further research into AMR dissemination are needed to address the human health, animal health and environmental concerns.

## Introduction

Antimicrobials, in the context of this review, include antibiotics, antiseptics and disinfectants. Antimicrobial resistance (AMR) among bacteria is an urgent global health issue. The World Health Organization (WHO) has deemed it “One of the greatest threats we face as a global community” (WHO, 2019). Approximately 700,000 people die from AMR bacterial infection each year, and, if no action is taken, this figure is predicted to increase to 10 million deaths per year by 2050 (WHO, 2019). The production of antibiotics by microorganisms is a natural process which is designed to kill or inhibit the growth of competing microorganisms. However, many bacteria have evolved diverse antibiotic resistance mechanisms as a basic survival component. Recent investigations have revealed that some antibiotic resistance mechanisms can also confer cross-resistance against antiseptics and disinfectants. This is a natural unavoidable evolutionary process as AMR genes have been found in remote environments with minimum human interaction, such as the Polar Regions (Nolan et al., 2023a). The rise in AMR is partially the result of inappropriate use of antibiotics. In some jurisdictions, healthcare providers do not restrict the use of antibiotics thereby preventing efficient monitoring of overall drug usage levels (Hartinger et al., 2021). Antibiotic mismanagement can occur in both human and agricultural (mainly animals) sectors resulting in the release of antibiotics and antibiotic residues into the environment (Food & Agriculture Organization of the United Nations, 2018; Maillard et al., 2020). This can create a selective pressure that favours more resistant bacteria, allowing these resistant organisms to multiply leading to greater dissemination of resistance genes (Daly et al., 2023). The consequence of the rise of AMR is increased hospital treatment time and cost, along with increased potential of mortality resulting in further strain on healthcare systems (Hartinger et al., 2021). This has led to the requirement for increased surveillance of AMR bacteria and AMR genes following the One Health approach set out by the WHO (Larsson et al., 2018; Tiwari et al., 2022; WHO, 2015). The One Health initiative integrates the human, animal and environmental sectors emphasising their interconnection. Surveillance in humans and animals has been well established, with environment surveillance a more recent effort towards monitoring AMR progression (Huijbers et al., 2019; WHO, 2015).

In recent years (Finley et al., 2013), the environment has been recognised for playing a major role as a source and dissemination of AMR. Wastewater treatment plants (WWTP) are seen as one of the major sources of antimicrobial resistance genes (ARGs) and bacteria. Antibiotics consumed by animals and humans, along with antibiotic-resistant gut microbes are excreted into the environment via faeces and urine (Karkman et al., 2019). Faecal pollution in the aquatic environment can occur from both identifiable (or point) sources such as wastewater treatment plants and septic tank effluents and non-identifiable (or non-point) sources such as agricultural runoff and animal defecation (Camiade et al., 2020; Flores et al., 2023; Ragot & Villemur, 2022). Rainfall events which enable faeces to enter the water systems are a key driver of dissemination (Ahmed et al., 2018; González-Fernández et al., 2021). The combination of the various sources of AMR highlights the complexity of tracking faecal pollution in the aquatic environment. Faecal pollution can have negative impacts on health, the economy and the environment showing their interconnectedness and validating the One Health perspective (WHO, 2015, 2023). As a result of faecal pollution, there is a deterioration in drinking water quality and a greater risk of waterborne disease outbreaks. This is due to increased exposure to possible pathogenic bacteria via ingestion, physical contact (dermal route) and accidental inhalation with AMR complicating treatment (Hinojosa et al., 2020; Valério et al., 2022). These factors contribute to an increase in the potential disease burden and raise the risk of transmission, resulting in increased treatment costs and deterioration of recreational water quality. This can cause temporary or permanent closure of recreational sites causing a loss of income (Díaz-Gavidia et al., 2022). The aim of this scoping review is to identify how faecal pollution contributes to AMR in surface water, what are current methods of identifying sources of faecal pollution and what are the most commonly studied ARGs.

## Materials and methods

No formal review protocol was registered; however, the PRISMA-ScR guidelines were followed as a framework, and the PRISMA-ScR checklist was completed to ensure transparent reporting. The following research questions were created to direct the review “how does faecal pollution contribute to antimicrobial resistance in surface water?” and “how can the source of faecal pollution in surface water be tracked?”.

There were no predetermined genes or locations targeted in the study, as one of the aims was to identify the most commonly studied genes. Search terms were decided after a discussion between authors and information specialists (Appendices (See Table 3)). The databases used to find literature were Medline Ovid® ALL 1946 to November 13, 2023, and Scopus®. From the search terms, a search string was created and adapted to each of the databases. All Medline searches were carried out using the mapped and unmapped feature (Appendices (See Tables 3 and 4) to find literature relevant to the terms. All Scopus searches were carried out using the TITLE-ABS-KEY feature of the database highlighting specific terms in the title, abstract and keywords. All relevant papers were exported to RefWorks®.

### Inclusion criteria

The study was conducted in November 2023, which covered the period from January 2020 to November 2023, to identify papers that were recent at the time; this was to ensure that the review captured recent developments and the latest advances in methods used to identify sources of faecal pollution and track ARGs in surface waters. Previous literature reviews have already covered pre-2020 developments in this field, so including earlier developments would lead to duplication (Fewtrell & Kay, 2015; Cho, S. et al., 2020; Mathai et al., 2020). Only articles published in English are considered.

Articles that included “antimicrobial resistance” and “aquatic environment” and mentioned “faeces” as a cause of deterioration of water quality were included. This included articles that mentioned “wastewater treatment plants/WWTPs”, “sewage treatment plants”, “septic tanks”, “antimicrobial resistant genes/ARGs”, “faecal indicator bacteria/species” (*E*. *coli*, streptococci,* Enterococcus* spp., including faecal indicator phyla Firmicutes) and articles that mentioned “microbial source tracking”. Only primary articles were accepted.

### Exclusion criteria

The study excluded articles that were about “groundwater”, articles outside the date range, articles that were looking at “faecal carriage”, “antibiotic pollution of aquatic environments” and analysis of other substrates such as “soil”, reviews, seminars and those that only mentioned one of the three topics under investigation.

Upon further screening, papers that did not identify the source through a scientific methodology such as source tracking or phylogenetic typing were rejected.

## Results

**Fig. 1:**
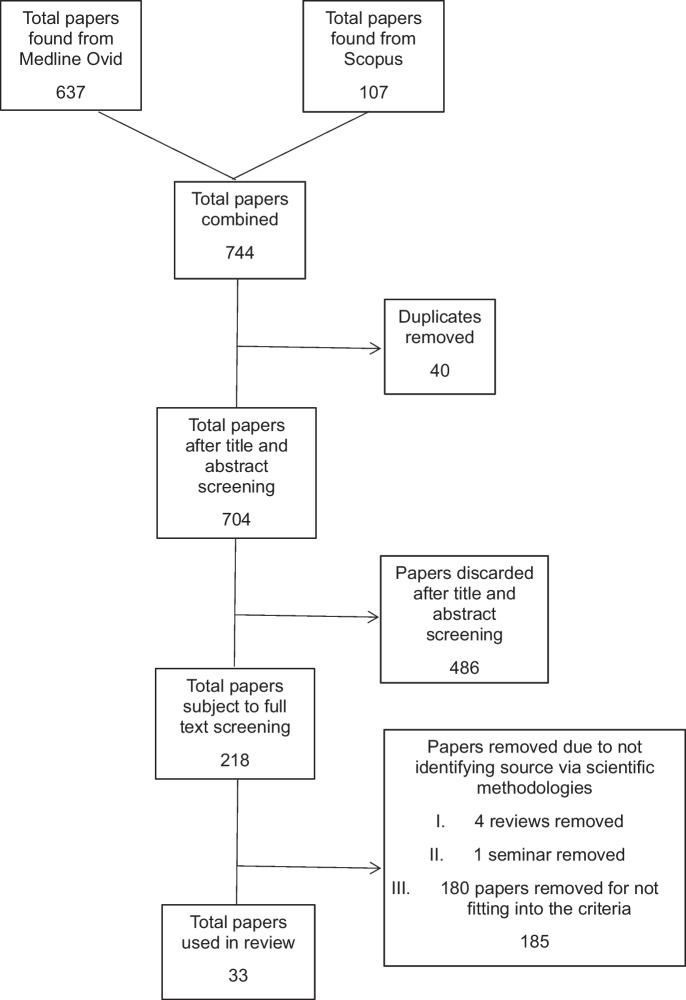
Identification of studies via databases

After conducting the searches on Medline Ovid® and Scopus®, a total of 637 and 107 papers, respectively, were identified, resulting in a combined total of 744 papers retrieved from both databases. On Medline Ovid® following initial title and abstract screening, a total of 171/637 were kept and exported to RefWorks, while the other 466 papers were discarded. On Scopus following initial title and abstract screening, a total of 87/107 papers were kept and exported to RefWorks, while the remaining were discarded; this gave a total of 258 papers. In all, 40 duplicate papers were removed, and this resulted in a total of 218 papers. The full screening of papers that (i) matched the inclusion and exclusion criteria and (ii) identified the source through means of tracking resulted in 185 papers being discarded, and a total of 33 papers were used for the review (Fig. 1).

After the full screening of 218 papers, a notable recurrent feature was observed. Papers such as Kimera et al. (2021) did not definitively identify the source and simply suggested that they are from human or animal sources based on the sampling location. Several studies such as Herrig et al. (2020) also suggested that applying source tracking could improve the study.

### Data characteristics

**Fig. 2:**
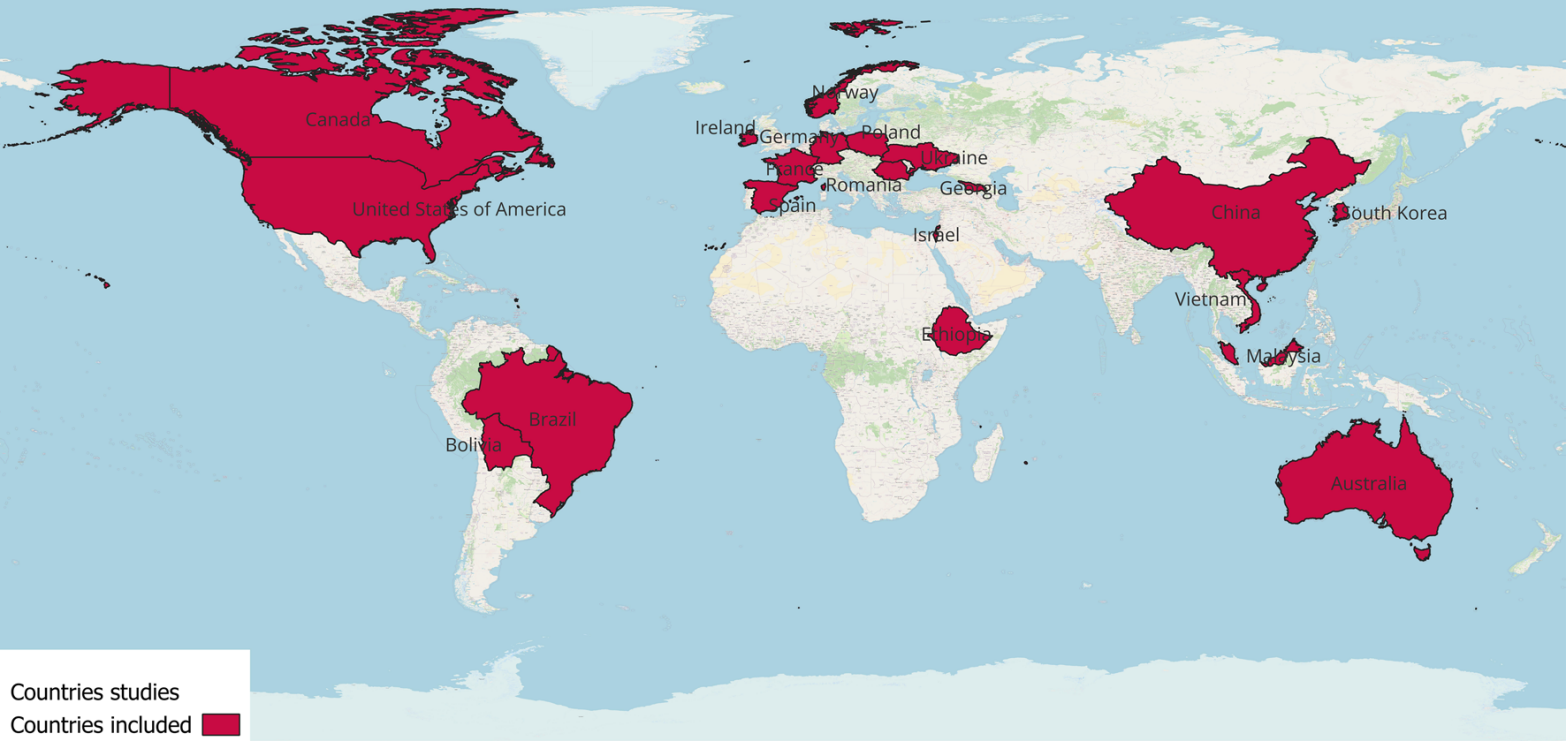
Countries/regions included by the selected research articles (an original image created with QGIS). Legend: This map displays a world map highlighting the countries/regions investigated in the selected studies, marked in red

The retained papers reported on studies that were conducted in six different continents are as follows: Europe (*n* = 11), Asia (*n* = 11), North America (*n* = 7), South America (*n* = 2), Africa (*n* = 1) and Oceania (*n* = 1). The countries/regions that the studies were conducted in were Norway, Germany and France (*n* = 2); Spain and Ireland (*n* = 4): Poland and Black Sea region (Romania, Ukraine and Georgia): Israel, Vietnam and China (*n* = 7); Korea, Malaysia and the USA (*n* = 6); and Canada, Brazil, Bolivia, Ethiopia and Australia (Fig. 2).

## Discussion

This scoping review was conducted to identify (i) how faecal pollution contributes to antimicrobial resistance in surface water, (ii) what are current methods of identifying sources of faecal pollution, and (iii) what are the most commonly studied ARGs. The search criteria ensured that relevant papers published between the years 2020 and 2023 were retrieved.

**Fig. 3:**
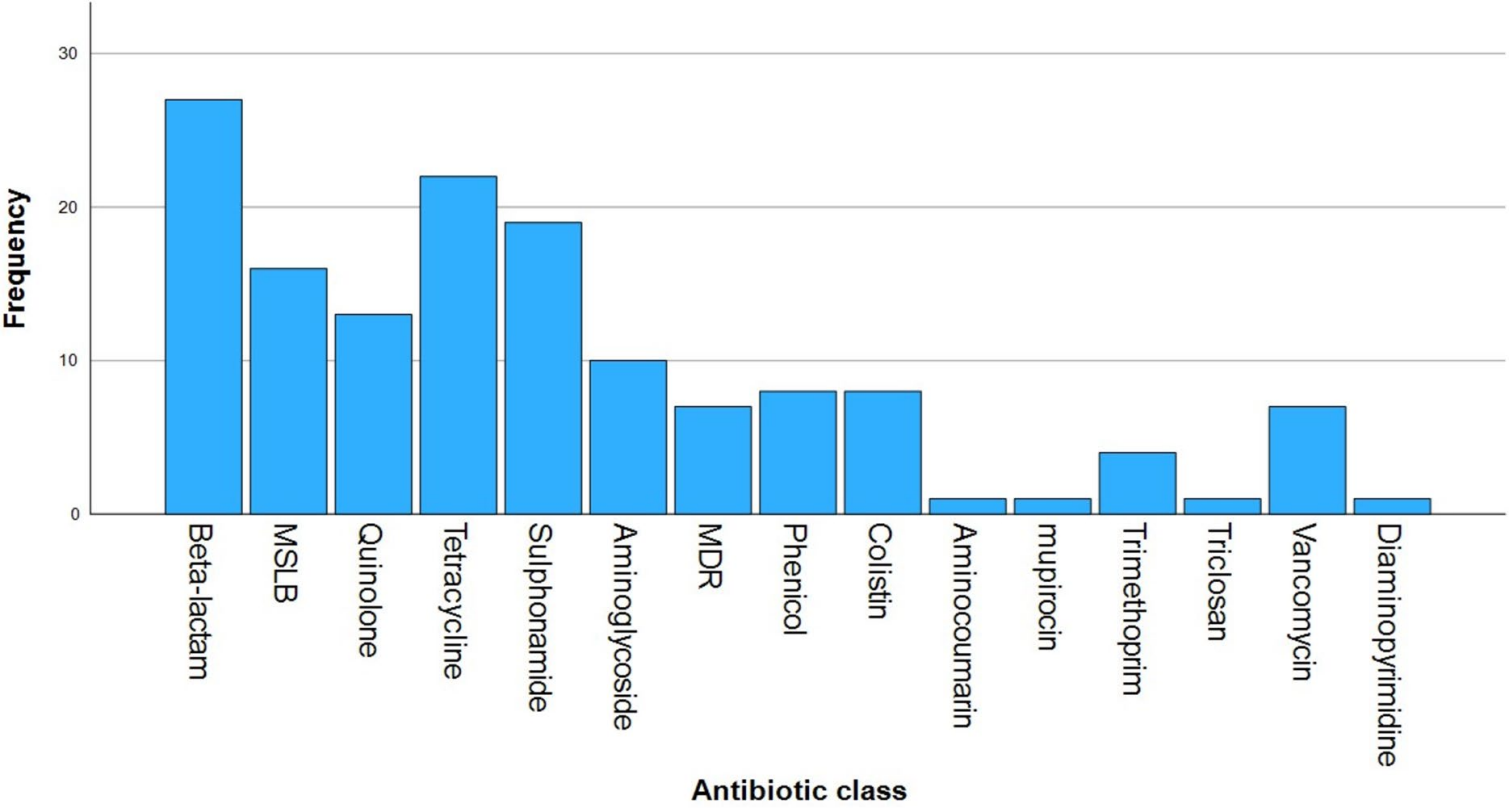
Frequency distribution of antibiotic classes studied by the selected research articles. Legend: This histogram displays the frequency of antimicrobial resistance genes categorised by antibiotic class, based on the data extracted from the 33 selected studies. Each bar represents the number of studies investigating a specific class of antimicrobial resistance gene defined by the antibiotic class for which these genes confer resistance

**Fig. 4:**
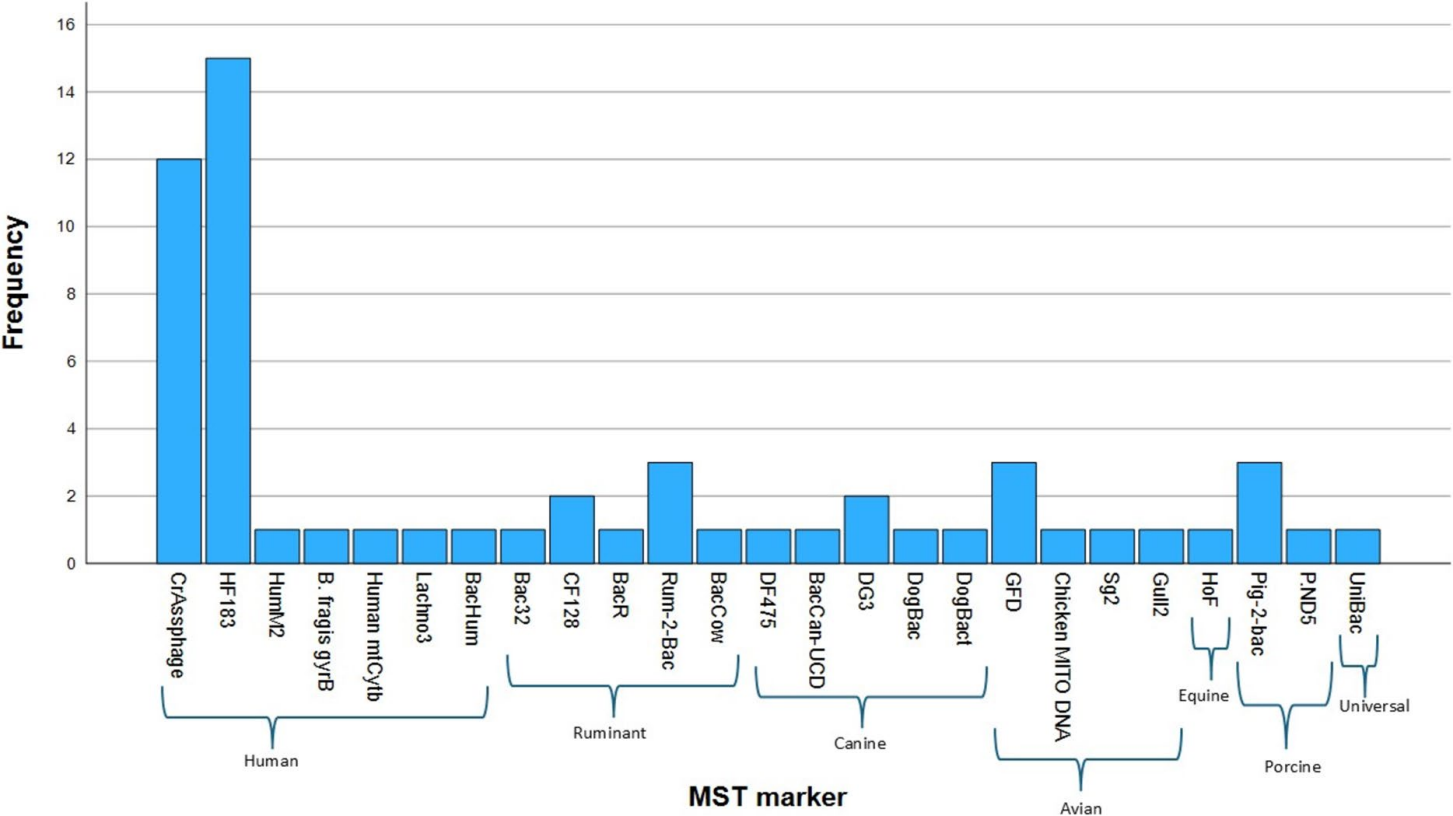
The frequency of MST markers studied by the selected research articles. Legend: This histogram displays the frequency of microbial source tracking markers utilised by the 33 selected studies. Each bar represents the number of studies utilising a particular marker, which are grouped by their specific source

The final selection for the review consisted of 33 papers that studied the impact of faecal pollution on the aquatic environment (Figs. 3 and 4). The studies were a combination of freshwater, marine water, wastewater and sediment highlighting the impact of faecal pollution in the deterioration of a variety of water sources used for drinking water, irrigation and recreation. The degradation of these water sources results in greater exposure to faecal pathogens leading to more waterborne disease outbreaks with AMR bacteria prolonging patient treatment (Valério et al., 2022). The majority of studies came from Europe and Asia comprising two-thirds of the entire review. Geographically spatiotemporal differences occur between countries with factors such as population density, sanitation infrastructure and environmental conditions that affect levels of faecal pollution. None of the reference material came from Northern Ireland and, by extension, the whole of the UK, identifying a potential gap in knowledge and indicating an opportunity for increased scientific contribution from the UK. A limitation of using two databases is that some relevant literature may not be covered due to publication bias and search inconsistencies. The identification of duplicates indicates overlap, which can lead to redundancy in the findings as well as add to the workload required to screen the data.

Only a few of the studies followed standard methods set out by governing bodies and organisations that provide guidelines for testing procedures such as EUCAST (2024), which were for antibiotics susceptibility testing, minimum inhibitory concentration and enumeration of faecal indicator bacteria (FIB) (European Union, 2006). Enumerating FIB is used to assess water quality and indicate potential faecal contamination, suggesting the potential risk of exposure to faecal pathogens (European Union, 2006). However, there are many limitations with just enumerating FIB; it does not identify the source, pathogenicity, virulence or resistance of strains (Williams et al., 2022). Therefore, studies could address this by utilising genetic analysis. A variety of genetic techniques are available: whole genome sequencing (WGS), Sanger sequencing, quantitative polymerase chain reaction (qPCR), metagenomics, multi-locus sequencing typing (MLST) and phylotyping, but there was no standardised method used in these studies (Table 1). However, all studies did carry out filtration of samples for the genetic analysis. A variety of methods to enumerate bacteria was used including the IDEXX Colilert test (Yakub et al., 2002), filtering samples and placing filters onto selective agar or adding drops/microlitres of sample directly onto agar. The majority of papers that did quantify bacterial counts used colony-forming units (CFU). However, a number of studies used most probable number (MPN) methods. Previous studies have shown that MPN figures are more variable and produce higher estimates than CFU (Gronewold & Wolpert, 2008; Cho, K. H. et al*.* 2010) highlighting the need for standardised methodologies to allow for comparison between results.

**Table 1:** The methodologies used and species investigated

Author	Methodology	Species (investigated)
Agramont et al. (2020)	Membrane filtration. qPCR was carried out to quantify ARGs, source tracking markers and MGEs	Not specified
Allsing et al. (2022)	Membrane filtration. Sequencing used to source track and ARGs. IDEXX Colilert test used to enumerate FIB	*E. coli*, *Stenotrophomonas maltophilia*, *Acinetobacter* sp., *Pseudomonas aeruginosa*, *Salmonella* sp.,* Arcobacter cryaerophilus*
Bagi et al*.* (2022)	Membrane filtration. Filters were placed onto selective media. Sequencing was employed. Sourcetracker2 was used to identify sources	Thermophile coliforms, faecal/gut (*Bacteroides*, Firmicutes, *Prevotella*, *Lactococcus*, *Ruminococcus*), environment (*Limnohabitans*, Sporichthyaceae, *Polynucleobacter* and *Arcobacter*)*
Chen, H. et al*.* (2020)	Shotgun sequencing was carried out. Raw reads were quality filtered which aligned with ARG and MGE structures. FEAST was employed to identify the sources	Proteobacteria, Bacteroidetes, Firmicutes, Actinobacteria and Chloroflexi*
Chen, Z. et al*.* (2023)	Membrane filtration. PCR targeting ARGs, MGEs, crAssphage and 16S rRNA	Compared gut flora and microorganisms in water* Human faeces/gut* (*Actinobacteria, Bacteroidetes, Firmicutes and Proteobacteria) Water (Bacteroidetes and Proteobacteria)
Christenson et al. (2022)	Membrane filtration. Membranes placed on selective media. AST using disk diffusion. ddPCR was utilised for MST. PCR used to identify ARGs	*E. coli*
Damashek et al. (2022)	Membrane filtration. qPCR was also used to for ARG and MST	N/A (faecal marker genes)
Guo et al. (2023)	Membrane filtration and culture. AST was performed. PCR was used to identify ARGs and Transferability of MCR genes. MLST along with WGS was performed. Phylogenetics was used to identify relationships between ARGs and to identify source	*E. coli* (recipient of plasmid), *K. pneumoniae*
Henriot et al. (2022)	Membrane filtration. Filters were placed onto selective media. Phylotyping was used to identify source of resistant strains and ARGs	*E. coli*, *K*. *pneumoniae* and *P*.* aeruginosa*
Hiruy et al. (2022)	50–100 µL was added onto selective agar and agar that contained ceftazidime, cefotaxime, ceftriaxone, aztreonam and fluconazole. Membrane filtration. Real-time PCR to confirm isolates and quantify16S rRNA. Sequencing to source track	*E. coli*, streptococci and faecal coliforms
Ho et al. (2021)	Membrane filtration. Filters were placed onto selective agar. AST using Kirby-Bauer disk diffusion and MICs were measured. PCR amplification was carried targeting 16S rRNA. A library was created. Sanger sequencing carried out. Real-time PCR was employed to identify human source *E. coli* and ARGs	Enterobacteriaceae and gram-positive *Staphylococcus* spp. and *Enterococcus* spp.
Hou et al. (2021)	Membrane filtration. PCR was used to amplify 16S rRNA and sequenced HT-qPCR was used to identify the FIB. Real-time PCR was used for ARGs and MGEs	Faecal (Bacteroidetesand Firmicutes)*
Kneis et al. (2022)	Membrane filtration. Sequencing via shotgun sequencing. 16S rRNA genes were scanned to identify bacterial and source track	Faecal (Bacteroidetesand Firmicutes)*
Leao et al. (2023)	Membrane filtration and filters placed on selective agar plates with or without cefotaxime. MALDI-TOF validates assumptions. Sequencing carried out. Bacterial community identification was carried out by ddPCR targeting 16S rRNA. qPCR was used to identify ARGs and source track	*E*. *coli* and coliform
Lee, K. et al*.* (2020)	Membrane filtration. Shotgun sequencing based on 16S rRNA was used to identify bacteria taxonomy and source track. qPCR was used to identify ARGs. AST being carried out via disk diffusion	Faecal/gut (Bacteroidetes, Firmicutes, Actinobacteria and Proteobacteria (Enterobacteriaceae, Pseudomonadaceae, Moraxellaceae and Aeromonadaceae))*
Lee, S. et al*.* (2020)	Membrane filtration and filters placed to selective agar. ddPCR was used to identify ARGs and source track. WGS to identify bacterial taxonomy	*E. coli*, environment (Thaumarchaeota), faecal/gut (Bacteroidetes, Proteobacteria, Actinobacteria and Firmicutes)*
Li et al. (2023)	Membrane filtration and cultured. AST was carried and identified MICs. WGS was carried. Phylogenetic analysis	*E. coli*
Liang et al. (2023)	Membrane filtration and filter membranes placed on selective agar. Real-time qPCR targeting 16S RNA was used to identify ARGs and faecal indicators	*E*. *coli*, enterococci, *Vibrio* spp., *Pseudomonas* spp.
Ma et al. (2023)	Membrane filtration. Sequencing was carried out to identify ARGs. FEAST was used to source track	Firmicutes*
Moretto et al. (2022)	Membrane filtration. qPCR was carried out targeting the 16S rRNA for MST. AST was carried out using disk diffusion. A MALDI-TOF was used to identify the species. PCR of resistant genes was carried out on the isolates	*E. coli*
Nguyen et al. (2021)	Membrane filtration and incubated on selective media. qPCR was used to quantify ARGs and quantify source tracking marker	*E. coli*
Niestepski et al. (2020)	Membrane filtration. The number of bacteria cells was counted using FISH and DAPI staining methods. qPCR was used targeting the 16S rRNA to quantify ARGs, identify species and source track	*E. coli* and *E. faecalis*
Nolan et al. (2023a)	Membrane filtration and filter membranes were placed on selective agar. qPCR was carried to quantify ARGs and source track	*E. coli* and enterococci
Nolan et al. (2023b)	Membrane filtration. qPCR was carried out targeting 16S rRNA	Somatic coliforms
Prekrasna et al. (2022)	Membrane filtration. qPCR (real-time) was carried out for ARGs and source track	Bacteroidetes
Reynolds et al. (2020)	Membrane filtration. Filters were placed on selective agar. qPCR was used to analyse ARGs and source track	*E. coli* and intestinal enterococci
Sala-Comorera et al. (2021)	Membrane filtration and filters were placed on selective media. qPCR was used to quantify the amount of ARGs and source tracking markers	*E. coli* and intestinal enterococci
Sanderson et al. (2020)	Isolates were selected and underwent WGS. AST using disk diffusion. MLST was used to obtain the phylogenetics of the isolates to identify ARGs, virulence and source tracking	*Enterococcus* spp*., E*. *faecalis*, *E*. *faecium*, *E*. *casseliflavus* and* E*. *gallinarum*
Sidhu et al. (2023)	Membrane filtration and filter membranes placed on selective media. MIC was identified using E-test strips for cefotaxime and cefepime along with disk diffusion. qPCR was used to source track and identify ARGs	*E. coli*
Thornton et al. (2020)	Membrane filtration and filters were placed on to selective agar. Metagenomic analysis from the filters was carried out to identify ARGs and source track. Kirby-Bauer method was followed for the enumeration and AST of bacteria. Disk diffusion was used to determine MIC	*E*. *coli*
Toubiana et al. (2021)	Membrane filtration and filters were placed on selective agar. Real-time qPCR was used to identify ARGs and source track	*E*. *coli* and intestinal *enterococcus*
Williams et al. (2022)	Membrane filtration. qPCR targeting 16S rRNA to source track and quantify ARGs, sequencing of the 16S rRNA. Bacteria enumeration was done using the following Australian standard	Enterococci
Zhang et al. (2022)	Membrane filtration. qPCR was used to source track targeting 16S rRNA and to quantify ARGs	*E. coli*

*qPCR* quantitative polymerase chain reaction, *ARG* antimicrobial resistant gene, *MGE* mobile gene element, *FIB* faecal indicator bacteria, *FEAST* fast expectation–maximization microbial source tracking, *PCR* polymerase chain reaction, *rRNA* ribosomal ribonucleic acid, *AST* antibiotic susceptibility testing, *ddPCR* droplet digital polymerase chain reaction, *MST* microbial source tracking, *WGS* whole genome sequencing, *MIC* minimum inhibitory concentration, *HT-PCR* high throughput-polymerase chain reaction, *MALDI-TOF* matrix-assisted laser desorption ionization-time-of-flight, *FISH* fluorescence in situ hybridization, *DAPI* 4′,6-diamidino-2-phenylindole, *MLST* multi-locus sequence typing.*Whole microbial community of sample was studied.

### Antimicrobial resistance genes

Antimicrobial resistant (AMR) strains of FIB were identified in all the studies. The isolation of AMR isolates was carried out using media that had been supplemented with the following antibiotics: aztreonam, cefotaxime, ceftazidime, ceftriaxone, ciprofloxacin, colistin, fluconazole and meropenem. Additional investigations included antibiotic susceptibility testing (AST) and minimum inhibitory concentration (MIC), with the resulting isolates of interest being subjected to genetic analysis. These antibiotics are commonly used, and their presence in the aquatic environment may suggest whether urban or agricultural activity is influencing the waterway. Aztreonam, cefotaxime, ceftazidime, ceftriaxone and ciprofloxacin are used as first-line antibiotics for human infection. While colistin and meropenem are last resort antibiotics; the authors may have also selected these antibiotics to highlight the increase in resistance to clinically important antibiotics.

The most common beta-lactamase ARGs studied were *bla*_CTX_ (*N* = 12), *bla*_TEM_ (*N* = 12), *bla*_OXA_ (*N* = 11) and *bla*_SHV_ (*N* = 10). This is concerning as *bla*_CTX_, *bla*_OXA_ and *bla*_SHV_ confer resistance to carbapenems which are clinically significant as a last resort antibiotic (Reynolds et al., 2020). Another common ARG within this group was *bla*_KPC_ (*N* = 8) which encodes for a carbapenemase. These enzymes have gained much global attention as they have a broad spectrum, not only inactivating carbapenem antibiotics but other clinically important beta-lactam antibiotics in both Gram-negative and Gram-positive bacteria (Chen, Z. et al*.* 2023). The predominant ARG studied encoding tetracycline resistance was the *tet* gene with the most common subtypes being *tet*A (*N* = 8), *tet*O (*N* = 7), *tet*Q (*N* = 5), *tet*W (*N* = 5) and *tet*X (*N* = 5). For sulphonamides, the most common resistance gene studied was *sul* with the most common subtypes being *sul*1 (*N* = 16) and *sul*2 (*N* = 7). This is concerning as tetracycline and sulphonamides are not just restricted to human use but also used in livestock and poultry production (Damashek et al., 2022; Ma et al., 2023). The presence of both tetracycline and sulphonamides in waterways may indicate the potential impact of agriculture on the local resistome of the aquatic environment, with the potential for specific ARGs to be used as markers and indicators for agricultural faecal pollution. The most common aminoglycoside resistance genes detected were *aph* (*N* = 8), *aad* (*N* = 7) and *aac* (*N* = 6). Aminoglycosides are first-line antibiotics in human medicine (Krause et al., 2016). The most frequent quinolone resistance genes studied were *qnr*S (*N* = 11) and *mfd* (*N* = 2). The most frequent MLSB-detected ARGs were *erm*B (*N* = 6), *erm*F (*N* = 6), *ere*A (*N* = 4) and *inu*B (N = 4). Quinolone and MLSB antibiotics are usually reserved as the alternative when first-line options are not effective (Pham et al*.* 2019; Pardo et al., 2020). Notable phenicol ARGs detected were *cat* (*N* = 4), *cml* (*N* = 3) and *flo*R (*N* = 3). The most frequent colistin resistance gene studied was *mcr* (*N* = 7). The common resistance genes studied for vancomycin were *van*A (*N* = 3), *van*B (*N* = 3) and *van*C (*N* = 3). Colistin antibiotics are a last resort in human medicine and if ineffective may result in a longer infection time and high mortality rates (Guo et al., 2023; Mull et al., 2020). The extensive list of ARGs studied highlights the scientific relevance of addressing resistance to antibiotics; however, from this list, there are some missing classes such as oxazolidinones and lipopeptides indicating a possible knowledge gap that needs to be addressed.

The resistance gene studied for aminocoumarin was *parY*; mupirocin was *ile*S1; triclosan was *Tri*C and diaminopyrimidine variants of *dfr*, *dfr*E, *dfr*F and *dfr*G. The few papers studying resistance in these antimicrobials indicate a possible knowledge gap, particularly for antimicrobials such as triclosan and diaminopyrimidine which are used in soap and haircare products (Alfhili & Lee, 2019; Garre et al., 2018; Vincenzi et al., 2019). A variety of multiple drug resistance (MDR) genes were analysed with the most prevalent being *qacEdelta*1 (*N* = 3). MDR occurs when bacteria are non-susceptible to at least three or more antibiotic classes (Ho et al., 2021). While *E*. *coli* is generally considered non-pathogenic, the majority of healthcare-associated infections are due to MDR pathotypes expressing extended spectrum beta-lactamases (ESBLs). These strains are commonly isolated from surface waters influenced by human activity including drinking water sources and recreational water (Sidhu et al., 2023).

Overall, the common occurrence of these ARGs across separate geographical locations highlights the dissemination of resistance to these clinically important antibiotics and the potential impact on mortality rates due to the rise in AMR-associated bacterial infections. There is currently no standard set of markers for the tracking of ARGs (Leao et al., 2023) indicating a gap in knowledge. However, just because these genes are present does not mean that they are being transcribed and biologically active. Only a few papers carried out a phenotypic investigation through AST and MIC assays. This emphasises the need for routine monitoring and surveillance of ARGs in the aquatic environment employing complementary methodologies.

### Mobile genetic elements

**Fig. 5:**
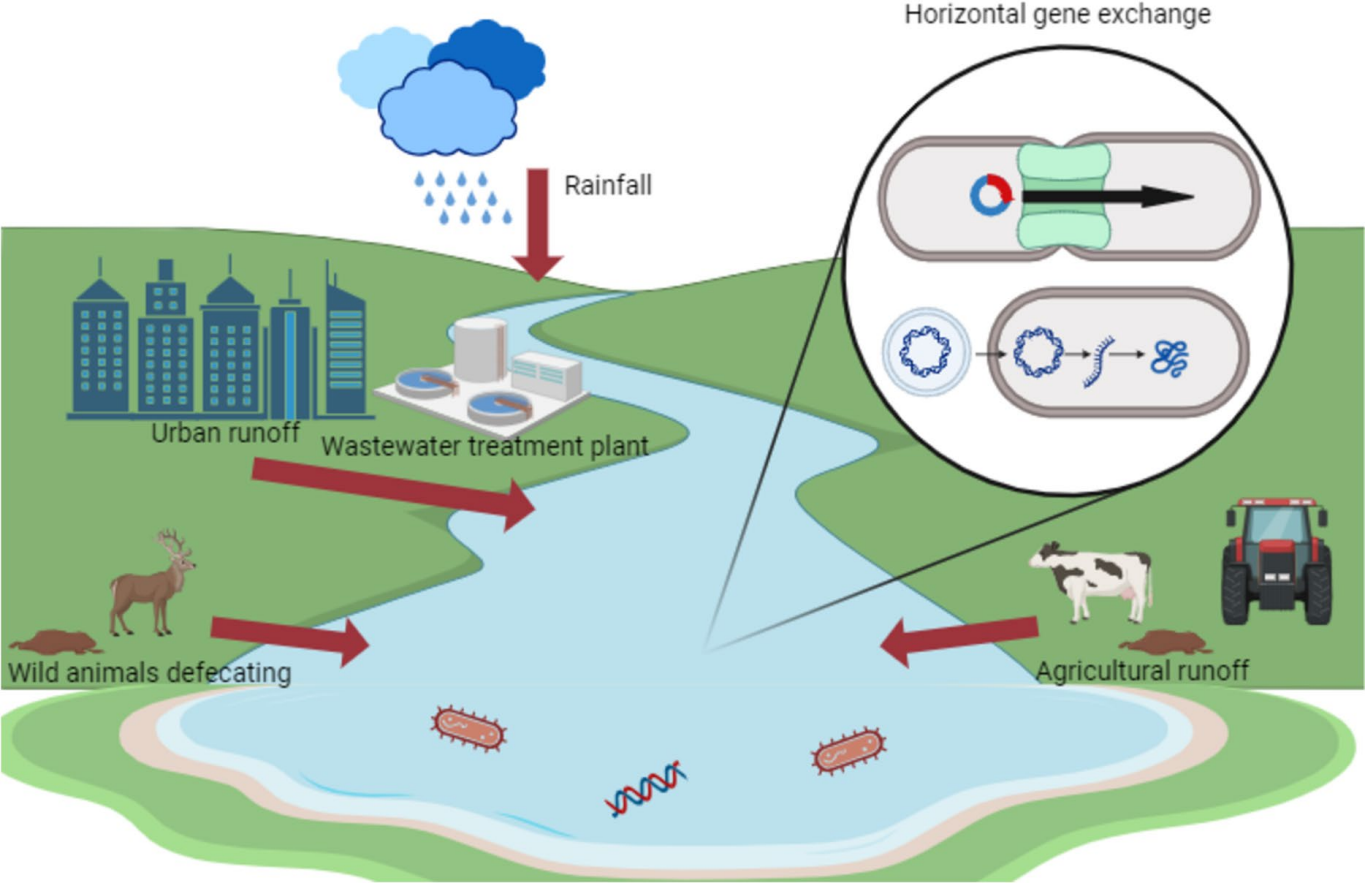
Sources of faecal pollution and how bacteria use mobile gene elements to disseminate antimicrobial resistance genes (an original image created with BioRender)

Mobile gene elements (MGEs) are genetic material that can aid the capture and transmission of exogenous genes; they include integrons, plasmids, transposons and genetic islands (Sanderson et al., 2020; Xie, 2021). MGEs aid in the dissemination of AMR between bacteria species by facilitating horizontal gene transfer (HGT) of ARGs (Fig. 5). Detection of high concentrations of MGEs suggests the possibility of significant HGT in a particular environment (Li, Y. et al*.* 2024). Overall, the papers identified various mobile genetic elements such as plasmids (*N* = 6), integrons (*N* = 13) and transposons (*N* = 6). These relatively low numbers from the studies suggest that there is a knowledge gap around their true distribution. This may be due to the difficulty in detecting MGEs as they contain many repetitive sequences, which may cause them to be misidentified as part of the non-coding regions. However, the increased availability of MGE sequences should facilitate the development of more focused markers (Xie, 2021).

### Source tracking markers

Microbial source tracking (MST) assays are used to assess water quality and identify possible sources of faecal pollution by targeting specific marker genes (Lee, S. et al*.* 2020). There are a variety of genetic markers available with the main types being those that target bacteria such as the HF183 *Bacteroides* 16S rRNA genetic marker that can be used to detect human faecal pollution in water environments (Lee, S. et al*.* 2020), those that target viruses such as crAssphage (Agramont et al., 2020) a bacteriophage highly specific to human gut (Stachler & Bibby, 2014) and those that target specific DNA such as mitochondrial DNA (Table 2). The link between a marker and source has been validated in previous studies. Accurate tracking of faecal pollution can measure the influence that human and animal faecal pollution has on ARGs in the aquatic environment (Chen, Z. et al*.* 2023). From the 33 studies, a variety of MST markers (Fig. 4) were used to identify human sources with the most commonly used marker being crAssphage (*N* = 13) and HF183 (*N* = 13). For ruminant sources, the most common is Rum2bac (*N* = 2). For avian sources, the most frequently used is GFD (*N* = 3), and for pig sources, the most common is Pig-2-bac (*N* = 3). The use of the same markers in different geographical regions may suggest that it may be possible in the future to have a single marker for each source to track faecal pollution worldwide enabling easier comparison of results.

**Table 2:** MST markers for different animal species used in source tracking in water sources

Microbial source tracking markers						
Human	Pig	Avian	Ruminant	Dog	Horse	Universal
crAssphage^1^ (Agramont et al., 2020)	Pig-2-bac^2^ (Christenson et al., 2022)	Sg2^2^ (Toubiana et al., 2021)	Rum-2-bac^2^ (Damashek et al., 2022)	BacCan^2^ (Hou et al., 2021)	HoF^2^ (Toubiana et al., 2021)	UniBac^2^ (Lee, S. et al*.* 2020)
HF183^2^ (Lee, S. et al*.* 2020)	P.ND5^3^ (Zhang et al., 2022)	GFD^2^ (Lee, S. et al*.* 2020)	Bac32^2^ (Moretto et al., 2022)	DG3^2^ (Williams et al., 2022)		
HumM2^2^ (Hou et al., 2021)		Chicken mitochondrial	CF128^2^ (Nolan et al., 2023a)	DogBac^2^ (Lee, S. et al*.* 2020)		
Lanchno3^2^ (Williams et al., 2022)		DNA^3^ (Damashek et al., 2022)	BacR^2^ (Prekrasna et al., 2022)			
crAssphage^1^ (Agramont et al., 2020)			BacCow^2^ (Zhang et al., 2022)			

^1 ^Virus target, ^2 ^Bacterial target, ^3 ^Mitochondria target

### Microbial community of faecal polluted water sources

Eight studies investigated the microbial community of samples and found that the community harboured multiple ARGs, with non-faecal indicator phyla such as Proteobacteria and Actinobacteria harbouring multi-drug resistance efflux pumps that are part of the resistance nodulation division (Bagi & Skogerbø, 2022). There is a correlation between the microbial community at a genus level and the profile of ARGs. Ma et al. (2023) found that the faecal indicator phyla Firmicutes harboured the largest possible amount of ARGs, one genus harbouring genes encoding resistance to MLSB (*erm*f, *mef*A, *erm*B, *lun*B and *erm*G), aminoglycosides (*aad*A and *aad*E), tetracyclines (*tet*M, *tet*36, *tet*O, *tet*W, *tet*32, *tet*O, *tet*X_2_ and *tet*44), chloramphenicols (*flo*R) and MDR (*qac*Edelta1). Similarly, Chen et al. (2020) found six MDR genes to be related to the indigenous microbial community. Hiruy et al. (2022) found that during the wet season, greater amounts of rainfall resulted in more runoff and soil erosion, which caused more soil-related genera, such as *Vicinamibacter* and *Legionella*, to be present in the aquatic environment potentially exchanging ARGs with the indigenous microbiome. This suggests that some ARGs originate from non-faecal bacteria and that there may be an exchange of ARGs between faecal and non-faecal bacteria.

Hou et al. (2021) found that in subtropical watersheds, MLSB and tetracycline resistance occur in bacterial species commonly found in the gut: *Bacteroides*, *Faecalibacterium*,* Clostridium*, *Blautia* and *Ruminococcus*, suggesting that faecal pollution aids in the dissemination of MSLB and tetracycline resistance. This highlights the potential role faecal pollution may have in influencing the ARG profile of watersheds, particularly urban waterways. In contrast, Ma et al. (2023) suggest that constant wastewater input contributes to the microbial community, impacting surface waters. However, this input does not affect the ARG profile, as horizontal gene transfer is not retained due to a change in environmental conditions. This change may be attributed to seasonal changes affecting pH, total organic carbon and heavy metal concentrations.

### Other microorganisms

From the selected studies, there was little investigation into other faecal-associated microorganisms such as protozoa (*N* = 2), bacteriophage (*N* = 3) and viruses (*N* = 1). These organisms play a significant role in disease and can act as a reservoir and disseminate AMR.

Like FIB, protists can be indicators of water quality; however, there have been few broad investigations of protists in urban environments (Lee, S. et al. 2020; Allsing et al., 2022). Both papers reported the presence of protozoa with Lee et al., (2020a, 2020b) profiling the protist community identifying potential for human disease outbreaks, while Allsing et al. (2022) detected the presence of protozoa and viruses that cause human disease, suggesting that the presence of any live virus or microbe may influence disease through activities such as swimming.

Only three studies investigated bacteriophages outside of the MST marker crAssphage. Nolan et al. (2023b) and Sala-Comorera et al. (2021) found ARGs to be harboured by the bacteriophages. While Sanderson et al. (2020) investigated HGT, this highlights the impact that bacteriophages play as a reservoir and a disseminator of AMR. Bacteriophages may also spread AMR from the environment to animals and humans with ingestion of contaminated water and shellfish (Nolan et al., 2023b). Overall, the low number of studies that investigated these microorganisms indicates a potential knowledge gap that should be addressed.

#### Correlation of mobile genetic elements with args

The most common MGEs studied were integrons notably class 1 integrons (*int*I1) being the most frequently studied. Markers associated with HGT such as integrons are usually found in areas where there is prevalent anthropogenic pressure particularly where wastewater is being deposited (Niestepski et al., 2020). For this reason, it has been proposed to use *int*I1 as an indicator of anthropogenic pollution (Nguyen et al., 2021). The integrons *int*I1, *int*I2 and *int*I3 are more common in terrestrial environments and less common in marine environments. Toubiana et al. (2021) found that *int*I2 and *int*I3 may be more specific as they were detected during peak faecal contamination during peak beach attendance, while *int*I1 may indicate other urban pollution. However, using *int*I1 as a marker may be unsuitable because it has the potential to contain ARGs, allowing for self-selection and potentially leading to challenges in distinguishing ARG dissemination from faecal pollution (Zhang et al., 2022). *int*I1 has also been identified to be utilised for the adaptation of other environmental stresses such as heavy metal in plankton-associated bacterial communities (Toubiana et al., 2021). Reynolds et al. (2020) highlight the complexity of integrons which are often associated with other MGEs resulting in further dissemination of ARGs that are commonly found in conjunction with *int*I1 such as sulphonamide resistance gene *sul*1. Zhang et al. (2022) support these findings with *sul*1 and *sul*2 having the strongest correlation with the abundance of *int*I1. Metagenomic analysis by Chen et al. (2020) found MGEs with the most associated resistance genes, the most common being *int*I1 and mupirocin resistance gene *ile*S1 and transposase *IS*91 contained *sul*2. These findings highlight the role that MGEs particularly *int*I1 have in shaping the resistome of the aquatic environment. The study also found that river sediment contributed significantly to the amount of MGEs indicating that the dissemination of ARGs within the river was largely connected to horizontal transfer promoted by the MGEs. Hou et al. (2021) found that the transposases *tnp*A-07 fuelled by the input of faecal bacteria are the keystone of HGT in an urban lagoon. Altogether, these studies highlight the significant role MGEs play in the persistence and dissemination of AMR within the aquatic environment introduced by anthropogenic pressures such as wastewater.

### The use of microbial source tracking to identify sources of faecal pollution and args

MST markers are a valuable tool for identifying the possible source of faecal pollution and the source of ARGs in the aquatic environment. Williams et al. (2022) found a significant correlation between the human faecal marker HF183 and *drf*A1, *sul*1, *qnr*S and *van*B resistance genes highlighting the link of raised ARG abundance and sewage input. The study by Chen et al. (2023) found that crAssphage abundance significantly correlated with the abundance of aminoglycoside (*aad*A, *aac*(6’)-Ib, *aad*A1, *aad*A2, *aph*A1, *aad*A5), MLSB (*erm*F), tetracyclines (*tet*X), quinolones (*aac*(6’)-Ib), phenicols (*cat*B3, *flo*R), sulphonamides (*sul*1, *dfr*A12), MDR (*tol*C) and beta-lactams (*bla*_OXA10_).

The study by Zhang et al. (2022) found a correlation between the carbapenem resistance gene *bla*_NDM-1_ and human markers (BacHum and CPQ056) and pig markers (Pig-2-Bac, P.ND5) suggesting that occurrence was due to combined human and pig pollution. A swine fever outbreak and the subsequent decrease in pig breeding resulted in a slight decrease in *bla*_NDM-1_ abundance. However, due to human faecal pollution being prevalent throughout the watershed, this decrease was not significant. Similarly, Damashek et al. (2022) found that the human faecal marker HF183 strongly correlated with carbapenem resistance genes *bla*_CTX-m1_, *bla*_SHV_ and *bla*_KPC_ in a multi-use watershed. The study found that the correlation between carbapenem resistance and ruminant and poultry markers was weaker compared to HF183; this suggests that while agricultural faecal pollution contributes to some of the ARGs, human faecal pollution remains the main source. These two studies highlight the usefulness of MST markers in identifying sources of faecal pollution and their contribution to the resistome of the aquatic environment.

A variety of factors influence the efficacy of MST markers, and differences exist between markers. Human markers, such as the *Bacteroides* marker HF183 and *Bacteroides* phage crAssphage, have different decay rates. crAssphage can be detected for more than 21 days, persisting longer than HF183, which can be detected for up to 10 days (Ballesté et al., 2019; Nolan et al., 2023b; Sala-Comorera et al., 2021). Therefore, crAssphage can travel further along the watershed (Zhang et al., 2022). This can influence the interpretation of results with prolonged persistence resulting in an overestimation of human faecal pollution, potentially giving false-positive results. Another important factor is the sensitivity and specificity of markers; many studies have a variety of results due to spatiotemporal differences arising between locations, countries and population. This can result in one MST marker being highly specific and highly sensitive in one area but not specific or sensitive in another area, e.g. in southern France, HF183 shows 56% sensitivity, while in California, USA, it is associated with 61% sensitivity (Toubiana et al. 2020) and in China, it has a 36% sensitivity (An et al., 2020). This can be attributed to differences in the gut microbiome, diet and sanitation infrastructure (Cao et al., 2013). This highlights the need for local investigations and ring trials to validate the use of markers. Some markers also showcase cross-reactivity between other species. Zhang et al. (2022) found that BacHum can cross-react with pigs and cattle. This cross-reaction resulted in BacHum correlating with the ruminant-associated ARG *tet*O, while the other human marker, CPQ056, did not. Cross-reactivity can result in false-positive and false-negative results; it is therefore important to consider this when selecting markers and highlights the importance of using multiple markers.

### The contribution of wastewater treatment to AMR dissemination

WWTPs play a significant role in contributing to faecal pollution of surface waters illustrated by several of the studies (Agramont et al., 2020; Niestepski et al., 2020; Sanderson et al., 2020; Nguyen et al., 2021; Bagi and Skogerbo 2022; Damashek et al., 2022; Hiruy et al., 2022; Kneis et al., 2022; Chen, Z. et al*.* 2023; Li, Q. et al*.* 2023; Leao et al., 2023). WWTPs harbour antibiotic residues and heavy metals creating an environment that selects for resistant bacteria. Chen et al. (2023) found that ARGs encoding resistance to tetracyclines, aminoglycosides and sulphonamides such as *tet*T, *tet*W, *tet*X, *str*A, *str*B, *sul*1 and *sul*2 (Fig. 3) were the most commonly found in WWTP samples. Agramont et al. (2020) found that aquatic lakes impacted by mining and wastewater effluent harboured an abundance of ARGs and *int*I1 indicating the impact of anthropogenic activity. This is consistent with Niestepski et al. (2020) findings on the impact of various wastewater effluent ranging from treated to urban wastewater on the aquatic environment. The study found a direct link with the contribution of wastewater to concentrations in *E. coli*, enterococci and *Bacteroides fragilis* and the abundance of ARGs. Damashek et al. (2022) extended the investigation by studying the entire watershed and found that non-point sources, such as aging septic tanks and sewage infrastructure, contribute significantly to the dissemination of AMR in aquatic environments. Consistent with this, Bagi and Skogerbø (2022) also found possible pathogenic strains of *Arcobacter* and *Bacteroides*, suggesting that continuous release of sewage into streams can result in a greater spread of sewage effluent towards beaches. Hence, the release of constant low-level wastewater may introduce potential pathogens into areas used for bathing.

There are many conflicting findings between the studies. The findings from Li et al. (2023) suggest that wastewater from WWTPs contributes to the release of ESBL *E. coli*. Strains from WWTPs exhibit genetic similarity to those found in the aquatic environment of the study, suggesting that the resistance genes along with the virulence factors and MGEs can disseminate from this source into water used for recreation, drinking water or irrigation in agriculture. Niestepski et al. (2020) findings also suggest that WWTPs are a source of contamination of river water introducing potentially pathogenic strains of *B. fragilis*, *E. faecalis* and *E. coli* which harbour ARGs, along with class 1 and class 2 integrases aiding the dissemination of AMR. Conflicting with these findings, a study by Kneis et al. (2022) found that ARGs that encode for resistance to trimethoprim (*dfr*B) and beta-lactams (*bla*_TEM_ and *amp*S) did not show a relationship with wastewater. Instead, these genes were most likely to be from the natural water microbiome independent of human influence or could be the result of non-point anthropogenic activities thereby emphasising the need for validated markers to unequivocally determine sources.

These studies confirm that wastewater significantly contributes to faecal pollution of surface waters through the release of ARGs and potentially pathogenic microorganisms, emphasising the public health risk along with the ecological concern. This highlights the need for standardised and robust wastewater management practices.

### The influence of the environment on faecal pollution

Environmental factors can influence faecal contamination and ARGs with temperature, pH and UV light exposure affecting the local microbial community (Leao et al., 2023). The presence of heavy metals like zinc can exert pressure selecting for resistant bacteria in soil which can be introduced into the aquatic environment via runoff; the presence of zinc and lead correlates with an increased abundance of genes encoding resistance to erythromycin (Agramont et al., 2020). To complicate this, further DNA from both humans and animals along with ARG decay rates can be influenced by the flow, dilution, sediment absorption and exposures such as UV light exposure (Chen, Z. et al*.* 2020). Li et al. (2023) found that the detection rate of ESBL *E. coli* decreased with higher levels of rainfall suggesting a dilution effect of rain to the river system. This is consistent with Hiruy et al.’s (2022) findings which found seasonal differences in bacterial communities in both wastewater influent and riverine bacterial communities, with the wet season causing a reduction in faecal streptococci and ESBL *E. coli*. Williams et al. (2022) found that 4 days of rainfall resulted in raised levels of human faecal markers. However, this may be due to the fact that *Bacteroides* can persist longer than FIB or that the genetic assays that quantify these markers detected nonviable bacteria which otherwise would not grow using traditional cultural-based methods. Stormwater infrastructure can play a significant role in faecal pollution and the subsequent dissemination of ARGs. Often, stormwater infrastructure is built near sewage infrastructure and can receive inputs from sewage during dry weather due to leaks and blockages, as well as overflow during rainfall events introducing untreated sewage into the aquatic environment (Williams et al., 2022). Rainfall can also introduce nutrients, silt and other pollutants that have built up during dry periods, and this can alter the composition of the local microbiota with pathogens that were present in the silt and sediment being introduced (Lee, S. et al*.* 2020). This change in species composition can also alter the resistome.

Differences in the antibiotics used can affect the local resistome; in Ireland, ciprofloxacin, sulphonamide, fluoroquinolone, tetracycline, trimethoprim and beta-lactam class antibiotics are some of the most used in both human and veterinary medicine (Nolan et al., 2023a, 2023b; Reynolds et al., 2020; Sala-Comorera et al., 2021). Beta-lactam antibiotics such as penicillins and cephalosporin are the most prescribed antibiotic in Irish hospitals accounting for 50% of prescriptions (Reynolds et al., 2020); this resulted in *bla*_TEM_ genes being the most abundant ARG found in all four papers followed by *bla*_SHV_ and *bla*_CTX_ which were also found in high abundance in two of the papers. All three ARGs also correlated with human faecal markers HF183 (Nolan et al., 2023a; Reynolds et al., 2020; Sala-Comorera et al., 2021) and crAssphage (Nolan et al., 2023b). However, these particular ARGs may have been selected because beta-lactams are the most common antibiotic used in the healthcare sector within Ireland and therefore are clinically important.

Animals can contribute to faecal pollution through fouling along waterways. Wild animals such as birds and deer overlap habitats with livestock where they may indirectly ingest AMR by grazing on faecal-contaminated pasture. Many of these animals often use wastewater effluent impacted sites for drinking water thereby ingesting AMR bacteria which can colonise the guts of these animals. They disseminate AMR bacteria by migrating to another area and defecating, this can be very prevalent with birds due to their ability to cover large distances. Some pathogenic strains of bacteria are often associated with animals; deer have been found to harbour virulent strains of *Salmonella enterica* serovar Typhimurium (Lee, S. et al*.* 2020). Dogs and birds have been known to harbour clinically important ARGs and therefore are a potential vector for the dissemination of AMR bacteria within the environment further increasing faecal contamination (Reynolds et al., 2020; Williams et al., 2022). The study by Williams et al. (2022) found that dog faeces and sewage on beaches increase the levels of *Enterococcus* by 95%. The persistence of ARGs within the aquatic environment at sites with minimal human impact suggests that wild animals such as deer and birds have a role in contaminating water sources (Nolan et al., 2023a). Domestic animals can also contribute; dogs are often walked in popular recreation sites such as beaches, hiking trails and rivers thus becoming a possible non-point source of faecal pollution (Reynolds et al., 2020). Tetracycline and sulphonamide resistance genes were some of the most abundant ARGs studied by the papers and could be attributed to both non-point and source point faecal pollution. As stated, these antibiotics are not restricted to human usage but employed in both livestock and poultry farming. Damashek et al. (2022) found that tetracycline resistance showed a strong correlation with areas within the watershed that had a greater agricultural influence. However, Zhang et al. (2022) showed that even though *tet*W and *tet*O are efflux pumps for tetracycline, the source and variation of locations were inconsistent. This may be because the occurrence and abundance of *tet*W were strongly associated with human faecal markers. While the occurrence and abundance of *tet*O being associated with pig, ruminant and poultry faecal sources, this suggests that different species may have different associated ARGs which could be utilised as a possible marker for agricultural and urban sources. Manure from these animals can be a source for these genes to enter the environment; antibiotic residues in livestock and poultry can be consumed from their meat (Ma et al., 2023) leading to exposure and selection for resistant bacteria within the intestinal tract. Thornton et al. (2020) found *int*I1to be present within the entire watershed despite different land uses and levels of pollution; sulphonamide resistance is often associated with *int*I1 which may indicate that it may be persistent within the natural environment due to other stresses.

## Conclusion

These studies highlight the complexity of ARG dissemination in the aquatic environment driven by faecal pollution from both urban and rural sources with human and animal inputs. Faecal pollution introduces different microorganisms particularly bacteria, which facilitate the dissemination of ARGs and MGEs into the environment across different geographical locations, emphasising the need for routine monitoring and surveillance of these genetic components within the aquatic environment. A major difficulty arises from investigators using different measurements highlighting the need for standardised methods to ensure comparability of interpretation and therefore to recognise emerging transnational issues. A mix of genetic and culture-based methods will aid in determining which ARGs are being transcribed. MST has proven to be a valuable tool in the determination of faecal pollution sources, but its’ efficacy depends on accounting for spatiotemporal dynamics indicating that local investigations should identify the best marker for usage and there are currently no standards set for the tracking of ARGs, which needs to be addressed. Robust wastewater management practices and future research into AMR dissemination and surveillance should address human health, animal health and environmental concerns with a focus on a One Health approach to encompass the multitude of factors affecting faecal pollution. A potential limitation of the study was the period (January 2020 to November 2023) of the literature identified for the scoping study. Some research laboratories were closed and/or working to minimal levels during the pandemic period, or their work was refocused to the national need for coronavirus SARS-CoV-2 testing. This may have restricted the collection of data during that time.

## Data Availability

No datasets were generated or analysed during the current study.

## Appendix

Table 3

**Table 3:** Search terms used to find literature

	Antimicrobial resistant terms	Faecal pollution	Surface water	Microbial source tracking
Search terms used (Scopus and Medline)	“antimicrobial resist*” or “antibiotic resist*”	“faecal pollut*” or “faecal contam*” or “fecal pollut*” or “fecal contam*”	“surface water*” or “bathing water*” or stream* or river* or lake* or wetland* or reservoir*	“microbial source tracking” or “bacterial source tracking” or “microbial tracing”
Medline unmapped search terms	antimicrobial resist* or antibiotic resist* antimicrobial resist* or antibiotic resist* antimicrobial resist* or antibiotic resist*	faecal pollut* or faecal contam* or fecal pollut* or fecal contam* faecal pollut* or faecal contam* or fecal pollut* or fecal contam* faecal pollut* or faecal contam* or fecal pollut* or fecal contam*	surface water* or bathing water* Surface water* or stream* or river* or lake* or wetland* or reservoir* surface water* or bathing water*	microbial source tracking or bacterial source tracking or microbial contamination tracing or microbial tracing
Medline mapped search terms	Drug Resistance, Multiple, Bacterial/ or Drug Resistance, Bacterial/ Anti-Bacterial Agents/ or Drug Resistance, Microbial/ or Drug Resistance, Bacterial/ Anti-Bacterial Agents/ or Drug Resistance, Bacterial/ or Drug Resistance, Multiple, Bacterial/	Feces/ or Sewage/ Escherichia coli/ Feces/ or Environmental Monitoring/ or Escherichia coli/	Rivers/ or Water Supply/ or Water/ Rivers/ Rivers/mi, vi [Microbiology, Virology]	Escherichia coli/ or Water Microbiology/ or Water Pollution/ or Environmental Monitoring/

Table 4

**Table 4:** Search query for Medline

1	(antimicrobial resist* or antibiotic resist*).mp. [mp=title, book title, abstract, original title, name of substance word, subject heading word, floating sub-heading word, keyword heading word, organism supplementary concept word, protocol supplementary concept word, rare disease supplementary concept word, unique identifier, synonyms, population supplementary concept word, anatomy supplementary concept word]
2	Drug Resistance, Multiple, Bacterial/ or Drug Resistance, Bacterial/
3	1 or 2
4	(faecal pollut* or faecal contam* or fecal pollut* or fecal contam*).mp. [mp=title, book title, abstract, original title, name of substance word, subject heading word, floating sub-heading word, keyword heading word, organism supplementary concept word, protocol supplementary concept word, rare disease supplementary concept word, unique identifier, synonyms, population supplementary concept word, anatomy supplementary concept word]
5	Feces/ or Sewage/
6	4 or 5
7	(surface water* or bathing water*).mp. [mp=title, book title, abstract, original title, name of substance word, subject heading word, floating sub-heading word, keyword heading word, organism supplementary concept word, protocol supplementary concept word, rare disease supplementary concept word, unique identifier, synonyms, population supplementary concept word, anatomy supplementary concept word]
8	Rivers/ or Water Supply/ or Water/
9	7 or 8
10	(microbial source tracking or bacterial source tracking or microbial contamination tracking or microbial tracing).mp. [mp=title, book title, abstract, original title, name of substance word, subject heading word, floating sub-heading word, keyword heading word, organism supplementary concept word, protocol supplementary concept word, rare disease supplementary concept word, unique identifier, synonyms, population supplementary concept word, anatomy supplementary concept word]
11	Escherichia coli/ or Water Microbiology/ or Water Pollution/ or Environmental Monitoring/
12	10 or 11
13	3 and 6 and 9 and 12
14	limit 13 to (english language and yr="2020 -Current")
15	(antimicrobial resist* or antibiotic resist*).mp. [mp=title, book title, abstract, original title, name of substance word, subject heading word, floating sub-heading word, keyword heading word, organism supplementary concept word, protocol supplementary concept word, rare disease supplementary concept word, unique identifier, synonyms, population supplementary concept word, anatomy supplementary concept word]
16	Anti-Bacterial Agents/ or Drug Resistance, Microbial/ or Drug Resistance, Bacterial/
17	15 or 16
18	(faecal pollut* or faecal contam* or fecal pollut* or fecal contam*).mp. [mp=title, book title, abstract, original title, name of substance word, subject heading word, floating sub-heading word, keyword heading word, organism supplementary concept word, protocol supplementary concept word, rare disease supplementary concept word, unique identifier, synonyms, population supplementary concept word, anatomy supplementary concept word]
19	Escherichia coli/
20	18 or 19
21	(Surface water* or stream* or river* or lake* or wetland* or reservoir*).mp. [mp=title, book title, abstract, original title, name of substance word, subject heading word, floating sub-heading word, keyword heading word, organism supplementary concept word, protocol supplementary concept word, rare disease supplementary concept word, unique identifier, synonyms, population supplementary concept word, anatomy supplementary concept word]
22	Rivers/
23	21 or 23
24	17 and 20 and 23
25	limit 24 to (english language and yr="2020 -Current")
26	(antimicrobial resist* or antibiotic resist*).mp. [mp=title, book title, abstract, original title, name of substance word, subject heading word, floating sub-heading word, keyword heading word, organism supplementary concept word, protocol supplementary concept word, rare disease supplementary concept word, unique identifier, synonyms, population supplementary concept word, anatomy supplementary concept word]
27	Anti-Bacterial Agents/ or Drug Resistance, Bacterial/ or Drug Resistance, Multiple, Bacterial/
28	26 or 27
29	(faecal pollut* or faecal contam* or fecal pollut* or fecal contam*).mp. [mp=title, book title, abstract, original title, name of substance word, subject heading word, floating sub-heading word, keyword heading word, organism supplementary concept word, protocol supplementary concept word, rare disease supplementary concept word, unique identifier, synonyms, population supplementary concept word, anatomy supplementary concept word]
30	Feces/ or Environmental Monitoring/ or Escherichia coli/
31	29 or 30
32	(surface water* or bathing water*).mp. [mp=title, book title, abstract, original title, name of substance word, subject heading word, floating sub-heading word, keyword heading word, organism supplementary concept word, protocol supplementary concept word, rare disease supplementary concept word, unique identifier, synonyms, population supplementary concept word, anatomy supplementary concept word]
33	Rivers/mi, vi [Microbial, Virology]
34	32 or 33
35	28 and 31 and 34
36	limit 35 to (english language and yr="2020 -Current")
37	25 or 26
38	14 or 37
